# Exploring Meta-Reasoning Propositional Confidence in Conspiratorial Beliefs and Socio-Cognitive Polarization

**DOI:** 10.1162/opmi.a.20

**Published:** 2025-08-29

**Authors:** Carola Salvi, Marta K. Mielicki, Alice Cancer, Paola Iannello, Tim George

**Affiliations:** Department of Psychological and Social Sciences, John Cabot University, Rome, Italy; SRI Education, SRI International, Menlo Park, CA, USA; Department of Psychology, Università Cattolica del Sacro Cuore, Milan, Italy; Department of Neuroscience and Cognitive Science, University of Maryland, College Park, MD, USA

**Keywords:** conspiracy theories, metacognition, problem solving, socio-cognitive polarization

## Abstract

Conspiracy theories have pervaded human thought across time and cultures, often emerging during crises such as the COVID-19 pandemic, where they influenced public behaviors and attitudes, notably in vaccine hesitancy. This research explores the metacognitive foundations of conspiracy beliefs, particularly focusing on how individuals monitor and assess their problem-solving processes. We propose that conspiracy beliefs are linked to high *propositional confidence*—often unsupported by accurate reasoning. Two studies were conducted to investigate the potential relationship between meta-reasoning inaccuracies (i.e., prospective confidence judgments and commission errors) during problem solving and conspiracy beliefs. Across two studies, we examine metacognitive markers of this overconfidence. Study 1 analyzes archival data from George and Mielicki’s (2023) to investigate how COVID-19 conspiracy beliefs are associated with initial judgments of solvability in solvable and unsolvable Compound Remote Associate (CRA) tasks. Study 2 examines the relationship between commission errors on Rebus puzzles and conspiracy beliefs, while also assessing *Socio-Cognitive Polarization* (SCP)—a construct encompassing ideological rigidity, intolerance of ambiguity, and xenophobia. Results show that SCP amplified the effects of commission errors on conspiracy beliefs, situating these cognitive patterns within socio-political contexts. These findings offer novel evidence that conspiracy beliefs are not merely a product of what people think, but how they think—underscoring the intertwined roles of flawed meta-reasoning and socio-political attitudes in sustaining conspiratorial worldviews.

*“Inasmuch as these witches are devilish and very crafty, truly they can infect with their poison whatever is on the earth that is corruptible and mortal.”*

Lambert Daneau, *De veneficiis* (1564), in *Witchcraft in Europe 400–1700: A Documentary History* ed. by Alan Charles Kors and Edward Peters (Philadelphia: University of Pennsylvania Press, 2001), p. 272.

## INTRODUCTION

From early belief in witchcraft and magic, conspiracy theories have been pervasive throughout human history, across cultures and different political, social, and psychological contexts (e.g., Lewandowsky et al., 2017; van Prooijen & Douglas, 2017). During the COVID-19 pandemic, conspiracy theories profoundly shaped public opinion, attitudes, and behaviors—most notably through widespread vaccination hesitancy—thereby highlighting the urgent need to understand the reasoning patterns underlying conspiratorial thinking.

The foundation of conspiracy beliefs lies in the way individuals process information and interpret events that occur in the world (Douglas et al., 2017, 2019; van Prooijen & van Vugt, 2018). Within this framework, conspiracy believers engage in a form of knowledge assessment that manifests as confidence, a feeling of rightness, and awareness of the validity/fallibility of their beliefs, which can be used as an internal control signal to guide behavior (Balsdon et al., 2020; Desender et al., 2018; Rollwage & Fleming, 2021). This meta-reasoning process is highly relevant to the investigation and understanding of conspiracy beliefs, which often thrive on individuals’ confidence that they possess hidden or privileged knowledge, leading them to believe in the existence of secret plots or cover-ups. Monitoring certainty (or uncertainty) during meta-reasoning offers insights into how effectively cognitive processes unfold, especially in contexts where unreliable heuristic cues can inflate confidence, as well as their success likability (Ackerman & Thompson, 2017; Lebuda & Benedek, 2023). Those who believe in conspiracy theories, indeed, often display high propositional confidence (i.e., certainty in a self-centered frame of reference; Fleming, 2024), overestimating the accuracy of knowledge and beliefs, and inflated reliability of their understanding of causality. This inflated confidence leads them to feel certain about the truthfulness of conspiratorial narratives, even when faced with contradictory evidence or a lack of credible support (Oettingen et al., 2022; Vitriol & Marsh, 2018).

When individuals possess high propositional confidence, they believe they have a deep understanding of a problem even if their knowledge is unsupported or flawed. Similarly, individuals who hold conspiracy beliefs tend to perceive patterns or connections between unrelated events, seeing hidden motives or orchestrated plots behind them (Whitson & Galinsky, 2008). This illusion of pattern recognition reinforces the belief in the conspiracy and reiterates their propositional confidence leading individuals to overestimate their ability to accurately interpret complex societal issues through conspiratorial explanations. These reasoning circularities are fostered by a tendency toward pattern-seeking behavior, and the perception of meaningful coincidences (a phenomenon also known as apophenia; Fyfe et al., 2008).

In this research, we seek to delve into the monitoring processes underlying COVID-19 conspiracy beliefs, by investigating initial judgment of solvability (Ackerman & Thompson, 2017) and commission errors^1^ as a measure of meta-reasoning^2^. We specifically adopt problem-solving paradigms that are neutral in social content or ideological constraints specifically to isolate and examine the pure assessment of knowledge strategies underlying metacognitive monitoring (i.e., subjective assessment of how well a cognitive task is, will, or has been performed; Ackerman & Thompson, 2017).

### How Might Conspiracy Beliefs Relate to Problem-Solving Meta-Reasoning?

Skilled problem solvers demonstrate high cognitive flexibility thus the ability to break free from rigid perspectives and approach problems from different angles. During meta-reasoning they actively explore alternative reasoning paths that can lead to viable solutions and are patient when faced with complex problems, understanding that finding the right solution may require time and perseverance (Iannello et al., 2025). Recent studies demonstrate that cognitive flexibility, as measured via problem-solving paradigms, translates into critical thinking skills and reflects individuals’ ability to navigate digital information with less polarized perspectives (Iannello et al., 2025; Salvi, Barr, et al., 2023; Salvi, Iannello, et al., 2023; Salvi et al., 2021; Zmigrod, 2020; Zmigrod et al., 2019). As evidenced by these new studies flexible thinking extends to various domains of social reasoning (Iannello et al., 2025; Salvi, Iannello, et al., 2023) including the capacity to critically question political ideologies (Salvi, Barr, et al., 2023; Salvi, Cristofori, et al., 2016).

In contrast, less skilled problem solvers may be more likely to believe they have found a solution to a problem without fully exploring all relevant information, even in the absence of an actual solution. This form of propositional confidence could potentially prevent them from thoroughly investigating the problem and thus identifying the most effective solutions. Indeed, prior work evidence this kind of inflated propositional confidence when measuring initial judgment of solvability, specifically in individuals who are highly receptive to bullshit (i.e., perceiving meaningless statements as profound; George & Mielicki, 2023), a feature which has been shown to relate to belief in conspiracies (Pennycook et al., 2015). Given that conspiracy theorists often perceive reality as containing hidden truths or unsolved problems, they may engage in complex reasoning processes, making the investigation of their meta-reasoning particularly relevant and informative.

Existing work exploring conspiracy belief and related constructs within problem-solving paradigms evidenced an inverse relationship between problem-solving accuracy and believing in fake news, overclaiming, conspiracy beliefs, bullshit receptivity (Cancer et al., 2023; Čavojová et al., 2020; George & Mielicki, 2023; Pennycook et al., 2015, 2020; Pennycook & Rand, 2019; Salvi, Barr, et al., 2023; Salvi, Iannello, et al., 2023; Salvi et al., 2021). These studies have used a variety of cognitive tasks to assess core problem-solving abilities, convergent creative thinking, and analytical thinking, such as the Compound Remote Associates (CRA) test, Rebus Puzzles, and the Cognitive Reflection Test (Bowden & Jung-Beeman, 2003; Frederick, 2005; MacGregor & Cunningham, 2008; Sebalo et al., 2023). Other recent work has also shown that higher problem-solving skills are negatively related to COVID-19 vaccine resistance, conservative attitudes, and religious fundamentalism (Cancer et al., 2023; Salvi, Barr, et al., 2023; Salvi, Iannello, et al., 2023; Salvi et al., 2021; Zmigrod, 2020; Zmigrod et al., 2018, 2019). However, the broader link between meta-reasoning during problem solving and conspiracy beliefs endorsement remains understudied. Thus, in the present investigation, we examine two aspects of meta-reasoning—initial judgment of solvability and commission errors that may underlie conspiracy beliefs—across two studies utilizing different problem-solving paradigms.

Building on Ackerman and Thompson’s (2017) meta-reasoning framework, we propose that the propositional confidence underlying conspiracy beliefs can be understood through a dual-process model. This framework distinguishes between two interacting systems that govern reasoning and belief formation:

**Monitoring (System 1)**: This system relies on fast, heuristic-based judgments to monitor one’s problem-solving progress. It generates a quick, intuitive propositional confidence—based on surface-level cues like familiarity or fluency rather than deep analysis. Conspiracy believers may rely more heavily on this system, leading to overconfident predictions of success, even when faced with unsolvable problems.
**Control (System 2)**: This system engages slower, more deliberate analytical reasoning, exerting cognitive control to evaluate and revise initial judgments. However, when individuals experience inflated propositional confidence from System 1, they may skip this controlled reasoning process—manifesting as commission errors—leading to an unwarranted belief that incorrect solutions are valid.

### Research Plan

Because conspiracy theorists often believe that hidden truths are waiting to be uncovered, they may be particularly prone to perceiving unsolvable or non-existent problems as solvable. Studying their meta-reasoning is therefore crucial, as they may exhibit overconfidence in their ability to find solutions—especially in contexts where no solution (or complot) actually exists. In our first study, we measure participants’ general initial judgment of solvability using both solvable and unsolvable CRA problems. This approach allows us to assess their ability to discriminate between problems that have a solution and those that do not, thereby providing insight into their level of propositional confidence. George and Mielicki (2023) showed people solvable and unsolvable CRA problems (participants were not told that some problems were unsolvable) and asked them to predict their likelihood of solving them. Individuals highly receptive to bullshit (i.e., those who rated nonsense statements as profound) reported higher problem-solving predictions, but actually solved fewer problems and were less capable of discriminating between solvable and unsolvable problems when making metacognitive judgments relative to individuals who were less receptive to bullshit. In the first study, we re-analyzed the data from George and Mielicki (2023), in which information about participants’ COVID-related conspiracy beliefs was collected but not analyzed, to examine how COVID-related conspiracy beliefs vary as a function of participants’ predicted and actual performance on CRA problems. Within this specific paradigm, we use the term initial judgment of solvability (Ackerman & Thompson, 2017) in general to refer to participants’ confidence in their ability to solve upcoming CRA problems. We hypothesized that higher initial judgment of solvability in response to unsolvable problems would be associated with stronger beliefs in COVID-related conspiracy theories.

Conspiracy thinking is associated with a tendency to overestimate the validity of one’s conclusions and to see meaningful patterns in ambiguous or unstructured information. As such, individuals with stronger conspiracy beliefs may be more prone to commission errors, as they are more likely to misidentify incorrect answers as correct, reflecting a broader inclination toward this kind of epistemic overconfidence. Thus, in our second study, we investigate the link between commission errors, defined as instances in which participants claim to have a correct solution to a problem despite providing an incorrect solution) and conspiracy belief. Differently from the first one, this study employs Rebus puzzles (e.g., MacGregor & Cunningham, 2008) to measure both problem-solving accuracy and the frequency of commission errors.

We reasoned that CRA problems primarily assess general verbal ability, specifically the capacity to link words and create compound associations and that their accuracy is easy to check. By contrast, Rebus puzzles rely more on interpreting spatial relationships and often have less immediately apparent solutions. These characteristics make them particularly well-suited for investigating commission errors, as they create conditions where individuals may be more likely to overestimate the correctness of their responses. We hypothesize that commission errors would be specifically associated with stronger beliefs in conspiracy theories. By measuring these errors, we aim to gain insight into how a misplaced sense of certainty—a core feature of conspiracy ideation—manifests in problem-solving contexts.

The second study also includes a measure of cognitive-to-social reasoning transfer, captured by the concept of Socio-Cognitive Polarization (SCP). SCP is a novel construct that reflects a tendency for inflexible thinking which extends to both cognitive and social reasoning. It encompasses dimensions such as conservative political ideology, absolutist thinking, intolerance of ambiguity, and xenophobia, offering a comprehensive indicator of rigidity (Cancer et al., 2023; Salvi, Barr, et al., 2023; Salvi, Iannello, et al., 2023; Salvi et al., 2021). The interplay between problem solving, propositional confidence, and belief in conspiracy theories is multifaceted, encompassing not just cognitive and metacognitive, but also socio-political dimensions of conspiracy theories. While our main aim is to investigate the cognitive processes underlying conspiracy theories free from specific ideological constraints, we cannot neglect the crucial role played by socio-cognitive factors in this complex interplay, especially for conspiracy beliefs related to COVID-19 (e.g., Fleming, 2024). Some scholars, indeed, view conspiracy theories as misguided attempts to explain complex events, while others emphasize their political utility, noting that they often serve as propaganda tools to advance extremist ideologies (Byford, 2011; Uscinski & Parent, 2014). Furthermore, research suggests that more dogmatic individuals are less responsive to metacognitive cues of uncertainty when seeking new information, compared to their less dogmatic counterparts, often skewing political, scientific, and religious discourse in the process (Fischer & Fleming, 2024; Schulz et al., 2020). Thus, we include the SCP measure in the second study to explore the interplay between socio-political constructs, and COVID-19 conspiracy beliefs.

In sum, we propose that SCP exacerbates propositional confidence by reinforcing rigid belief structures that impair effective monitoring and control. Individuals with high levels of socio-cognitive polarization may:

**Strengthen System 1 reliance**: Rigid beliefs may heighten reliance on heuristic, pattern-seeking processes, leading individuals to interpret ambiguous information as supporting their pre-existing worldview.
**Weaken System 2 engagement**: Strong socio-political identities may create resistance to analytical reasoning and correction, as disconfirming evidence is more likely to be rejected to protect one’s ideological stance.

This interaction between meta-reasoning and socio-political polarization provides a novel lens to explain why conspiracy believers tend to exhibit overconfidence in their problem-solving abilities, particularly when faced with unsolvable tasks, and why they persist in these beliefs despite contradictory evidence. Our model underscores that addressing conspiracy beliefs requires more than simply improving analytical reasoning skills. It also calls for interventions that consider the broader socio-political environments that reinforce heuristic thinking and hinder metacognitive control processes.

## STUDY 1

In the first study, participants attempted both solvable and unsolvable CRA problems, and they were unaware of this distinction. This design allowed us to assess how individuals’ initial judgment of solvability might relate to their tendency to believe in conspiracy theories. Participants made predictions of their likelihood of solving CRA problems prior to actually attempting them. Thus, in Study 1, we included both solvable and unsolvable problems to disentangle metacognitive inaccuracies from accurate self-assessment. While our primary focus is on the initial judgment of solvability—the overconfident belief that an unsolvable problem is on the verge of being solved—the inclusion of solvable problems provided a necessary baseline, allowing us to assess participants’ ability to calibrate their confidence based on problem solvability.

Based on the existing literature on metacognitive biases and conspiracy beliefs, we hypothesized that:

H1: Individuals with a higher initial judgement of solvability on CRA problems, particularly unsolvable ones, will show greater endorsement of COVID-19 conspiracy beliefs.
H2: Based on prior research (e.g., Swami & Furnham, 2014; van Prooijen & Douglas, 2017), we expected actual problem-solving accuracy to relate to lower conspiracy beliefs.
H3: The relationship between problem-solving predictions and conspiracy beliefs will be stronger for unsolvable problems compared to solvable problems.

By examining these relationships in the context of a major global event (COVID-19), we aimed to enhance the ecological validity of our findings and contribute to understanding how conspiracy beliefs form and persist in times of crisis. This study set the stage for a more comprehensive examination of cognitive, metacognitive, and socio-cognitive factors in Study 2.

### Methods

#### Participants.

Participants (*M*_*age*_ = 35.93, *SD* = 11.80, 61% female) consisted of 100 U.S. individuals from Amazon’s Mechanical Turk. Data collection took place during May and June of 2020 as part of a broader study (George & Mielicki, 2023). As part of that study, data quality was ensured both through an initial attention screening, as well as through several indices of engagement that had to be met for inclusion in analyses (e.g., minimum response times, minimum number of responses provided, etc.). All participants provided an agreement to participate and were compensated with $1.75.

#### Materials.

A set of 12 CRA problems was used (see Supplementary Materials), with six solvable and six unsolvable. The problems were drawn from the norms of Bowden and Jung-Beeman (2003). The unsolvable problems were created by re-combining words from other CRA problems from these norms. The unsolvable problems were designed so that they would not appear immediately different from the solvable problems. An example of a solvable problem is *hound*, *pressure*, *shot* (solution: *blood*). An example of an unsolvable problem is *cone*, *aid*, *preserve* (no solution). See also Lauterman and Ackerman (2024) for a similar use of CRAs and initial judgment of solvability.

To assess COVID-based conspiracy beliefs, a six-item questionnaire was created in which participants indicated their agreement on a 1–5 scale with statements such as “The coronavirus pandemic is being exaggerated as a way to control people and limit individual freedoms.” This format was modeled after the Generic Conspiracist Beliefs (GCB) scale (Brotherton & French, 2014). The content of the questions was created based on dominant conspiracy theories at the time as determined by a web search (drawing dominantly from pewresearch.org, cvoid19misinfo.org, and allieanceorscience.org). Reliability was very good (Cronbach’s *α* = .94), and the average agreement was computed for each participant.

#### Procedure.

Participants were first instructed about the CRA and were provided an example problem and two practice problems with feedback. They were instructed that they would be given 15 seconds to solve each problem. Next, they were instructed that prior to attempting to solve the problems, they would first predict the likelihood of a solution. This followed Storm and Hickman’s (2015) procedure. Participants gave their predictions along a 0 (0% likelihood of solving) to 10 (100% likelihood of solving) scale, and they had a time limit of 5 s before automatically advancing to the next item. The 12 problems were presented sequentially in a random order for participants to complete their predictive rating. Participants were not informed that some of the problems were unsolvable.

After completing their predictions, participants actually attempted to solve the CRA problems (both solvable and unsolvable). The problems were presented sequentially in a random order along with a text response field. Participants had a time limit of 15s per problem before automatically advancing to the next problem, which is a duration consistent with other prior studies (Bowden & Jung-Beeman, 2003). After solving the CRA problems, participants completed the COVID conspiracy scale along with other demographic questions such as age, gender, race, and educational background.

### Results

Data analysis was performed using Jamovi version 2.6 for traditional analyses, and R was used for mixed effects models. Significance levels were set to *p* < 0.05. Overall, participants predicted a greater likelihood of solving the solvable CRA problems (*M* = 5.25, *SD* = 2.30) than the unsolvable problems (*M* = 4.45, *SD* = 2.23), *t*(99) = 5.15, *p* < .001, *d* = 0.52. For the six solvable CRA problems, participants solved an average of *M* = 2.44 (*SD* = 1.78) problems.

Correlations are displayed in Table 1. The most notable findings from this table are that COVID conspiracy beliefs were associated with higher predictions of CRA performance both for unsolvable problems, and solvable problems. COVID conspiracy beliefs were also negatively related to CRA solution accuracy.

**Table 1.** Correlation Matrix of Measures Used in Study 1.

**Variable**	**1**	**2**	**3**	**4**
**1. CRA solvable prediction**	–			
**2. CRA unsolvable prediction**	0.764***	–		
**3. COVID conspiracy**	0.188	0.376***	–	
**4. CRA accuracy**	0.081	−0.218*	−0.432***	–

* *p* < .05.

** *p* < .01.

*** *p* < .001.

**Figure 1.**
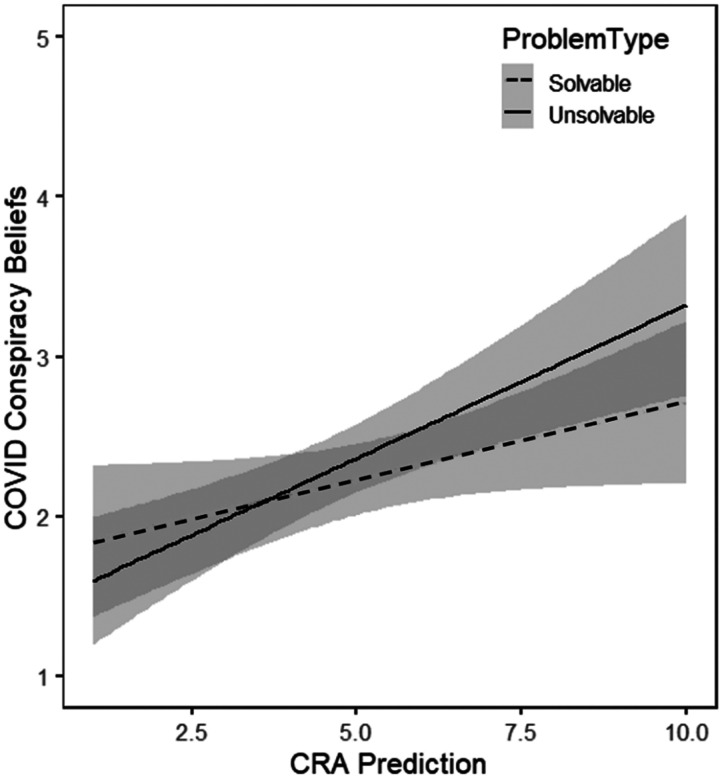
The relationship between CRA prediction magnitude and COVID conspiracy beliefs. Shaded areas represent 95% confidence bands.

Next, a regression model using COVID conspiracy beliefs as the outcome variable, and participants’ metacognitive predictions for solvable CRA problems and unsolvable CRA problems were entered as predictors^3^ (Figure 1). In this model, only predictions for the unsolvable problems emerged as a significant predictor of COVID conspiracy beliefs, *b* = 0.28, *SE* = 0.07, *t* = 3.89, *p* < .001. In contrast, predictions for solvable problems were not predictive of conspiracy beliefs, *b* = −0.12, *SE* = 0.07, *t* = −1.66, *p* = .100.

**Figure 2.**
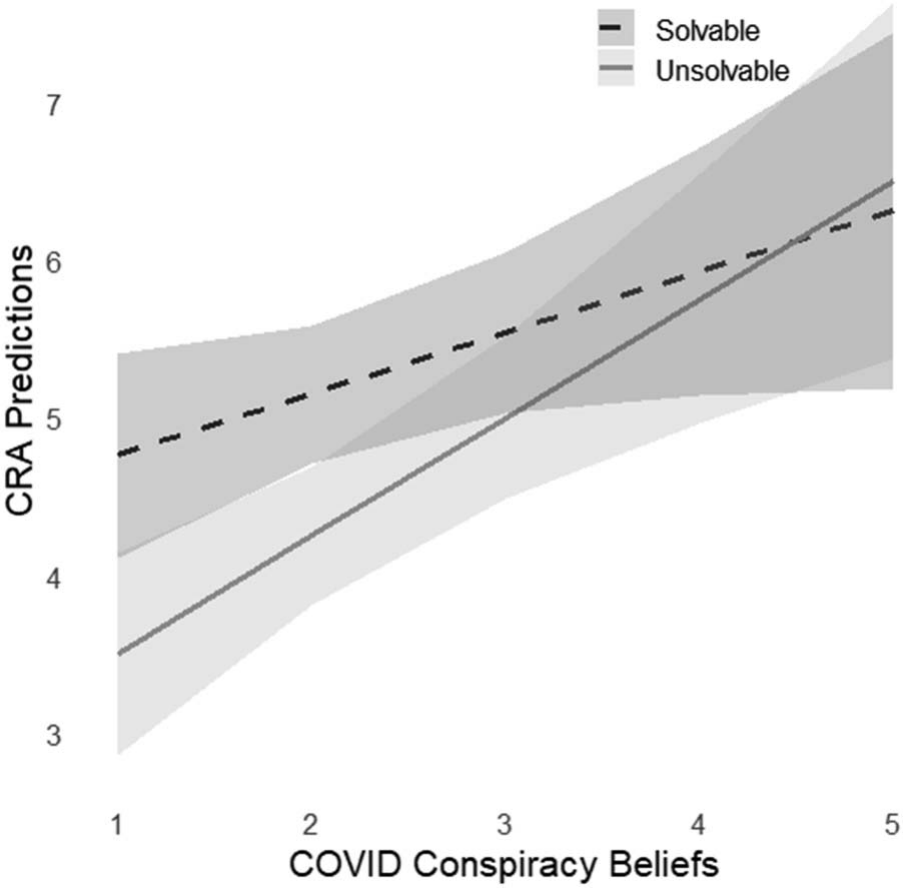
The relationship between COVID conspiracy beliefs and initial judgment of solvability for solvable and unsolvable CRAs. Shaded areas represent 95% confidence bands.

An additional analysis examined participants’ ability to discriminate solvable and unsolvable problems as a function of COVID-19 conspiracy beliefs. A regression model was conducted entering COVID Conspiracy Beliefs, Problem Type (solvable vs. unsolvable), as well as the COVID Conspiracy × Problem Type interaction as predictors, with CRA Prediction as the outcome variable. This resulted in a main effect of Conspiracy Beliefs, *b* = 0.39, *SE* = 0.19, *t* = 2.00, *p* = .04, a main effect of problem type, *b* = −1.63, *SE* = 0.34, *t* = 4.78, *p* < .001, as well as a significant interaction, *b* = 0.36, *SE* = 0.13, *t* = 2.69, *p* = .008. As shown in Figure 2, this interaction indicates that higher COVID Conspiracy Beliefs were associated with less discrimination of solvable and unsolvable problems in terms of the magnitude of their predictions of solving likelihood.

Lastly, we tested the relationship between participants’ CRA predictions and actual solving likelihood (for solvable problems only) as a function of both COVID Conspiracy Beliefs and problem solvability. We employed a logistic mixed-effects model entering CRA Predictions, COVID Conspiracy Beliefs, and their interaction as fixed effects, with problem-solving accuracy as the outcome variable. By-participant and by-item intercepts were entered as random effects. This resulted in a main effect of CRA Predictions, *b* = 0.24, *SE* = 0.09, *z* = 2.65, *p* = .008, with higher predictions being associated with higher solving accuracy, and a main effect of COVID Conspiracy Beliefs, *b* = −.60, *SE* = 0.26, *z* = −2.30, *p* = .022, with higher conspiracy beliefs associated with lower problem-solving accuracy. The interaction was not significant, *b* = −0.02, *SE* = 0.04, *z* = −0.52, *p* = .601. This indicates that while higher conspiracy believers were poorer at problem solving, both low and high conspiracy believers’ predictions were relatively metacognitively accurate (as indicated by subsequent solving likelihood) when it came to solvable problems.

### Discussion

These results show that, overall, participants were more confident in their ability to solve those CRA problems that were actually solvable, compared to those that were unsolvable, which is in line with Ackerman and Beller (2017). We additionally found that the strength of people’s initial judgment of solvability (see H1) was predictive of their belief in COVID-based conspiracy theories. The results of the regression model indicated that this effect was mainly for predictions for unsolvable problems (see H3). Furthermore, people who showed less differentiation in their confidence ratings between solvable and unsolvable problems (i.e., those who gave judgments of similar magnitude across both types) tended to endorse conspiracy beliefs more strongly. This supports the idea that inflated initial judgments of solvability are related to conspiracy beliefs. Finally, problem-solving accuracy was negatively related to conspiracy beliefs: individuals who solved more problems correctly were less likely to endorse conspiratorial narratives (see H2).

Higher initial judgment of solvability on solvable problems may, in some cases, reflect accurate self-assessment rather than overconfidence. To account for this, we examined whether participants’ predictions aligned with their actual performance on solvable trials. This allowed us to differentiate between participants who demonstrated calibrated confidence (i.e., high predictions paired with higher likelihood problem solving) versus those exhibiting inflated confidence (i.e., high predictions but low success rates). We found that while predictions on solvable problems correlated with performance—suggesting accurate self-assessment in general—predictions on unsolvable problems remained disproportionately high among conspiracy believers. This supports the interpretation that high conspiracy believers are more susceptible to a false sense of future problem-solving success. By using both solvable and unsolvable trials, we provide a clearer picture of how accurate self-assessment diverges from overconfidence, reinforcing the argument that conspiracy beliefs are linked to a persistent miscalibration of metacognition.

We acknowledge that while this re-analysis study is in line with our prediction it was only exploratory and is not sufficient to conclude that believing in conspiracy theories is *per se* associated with metacognitive inaccuracies. First, we only used a limited set of 12 CRA problems, and second, conspiracy theories during the COVID-19 pandemic were extremely volatile and tied to several factors. Thus, to strengthen our findings we designed a second experiment to examine whether the results would hold for a different metacognitive inaccuracy, specifically prospective confidence judgments and a different problem-solving task. In addition, we collected data on people’s levels of SCP to explore the relationships between socio-political dimensions of conspiracy belief and metacognitive inaccuracies in problem solving performance.

## STUDY 2

While Study 1 provided initial evidence for a relationship between conspiracy beliefs and propositional confidence in problem solving (i.e., general initial judgment of solvability), Study 2 aimed to extend these findings and explore the broader socio-cognitive context in which conspiracy beliefs emerge. Specifically, we sought to investigate how problem-solving abilities, (specifically, solving accuracy and commission errors), and socio-cognitive factors interact to influence belief in conspiracy theories. In Study 2, we employed Rebus puzzles to assess problem-solving accuracy and participants’ commission errors after claiming to have found a solution. This approach allowed us to examine not just problem-solving performance, but also participants’ propositional confidence in their solutions—a key aspect of metacognitive reasoning that we hypothesized would be linked to conspiracy beliefs. Importantly, Study 2 introduced the concept of Socio-Cognitive Polarization (SCP) as a potential mediating factor. SCP encompasses xenophobia, absolutism, and conservatism—factors that have been increasingly recognized as relevant to the formation and maintenance of conspiracy beliefs (e.g., van Prooijen & van Vugt, 2018; Zmigrod et al., 2019).

We included SCP for several reasons:

1. *Cognitive-social reasoning transfer*: Recent research suggests that cognitive flexibility in problem solving may extend to flexibility in social and political reasoning (Iannello et al., 2025; Salvi, Iannello, et al., 2023). SCP provides a framework to examine this potential transfer.
2. *Underlying cognitive mechanisms*: By including SCP, we can explore how cognitive processes interact with social and political attitudes to influence conspiracy beliefs while employing content-neutral problem-solving tasks that allow for a more precise investigation of underlying cognitive processes independent of specific contextual or ideological constraints.
3. *Multifaceted nature of conspiratorial thinking*: Conspiracy theories often involve not just cognitive biases (Teovanović et al., 2025), but also social and political elements. SCP allows us to capture these broader dimensions.
4. *Potential moderating role*: We hypothesized that SCP might moderate the relationship between problem-solving abilities and conspiracy beliefs, providing a more comprehensive model of how these factors interact.
5. *Addressing limitations of self-report measures*: By using SCP alongside objective cognitive tasks, we aim to overcome some of the limitations associated with relying solely on self-report measures of ideological thinking (Iannello et al., 2025; Zmigrod, 2020).

In sum, building on the findings from Study 1 and incorporating the concept of Socio-Cognitive Polarization (SCP), we hypothesized that:

H1: Higher problem-solving accuracy on Rebus puzzles would be negatively associated with conspiracy beliefs.
H2: Higher commission errors in Rebus puzzle solving would be positively associated with conspiracy beliefs.
H3: SCP would moderate the relationships between problem-solving abilities (both accuracy and commission errors) and conspiracy beliefs.

### Methods

#### Participants and Data Cleaning.

Data were collected in the United States from February to June 2021. CloudResearch was used to distribute the link to a Qualtrics-hosted online survey via Amazon’s Mechanical Turk (MTurk) platform. To ensure data quality, only pre-vetted workers who had demonstrated attention and engagement were allowed to participate. Only people older than 18 who lived in the United States and spoke American English as their first language were eligible for the study. The study only accepted participants who passed the initial screening. The hits and qualifications required by MTurk were used to select subjects. Participants received a total of $4 upon completion of the study, which took approximately 45 minutes to complete (with a median completion time of 42.3 minutes). All participants gave written informed consent, and the University of Texas at Austin Institutional Review Board approved the study.

A sample of 350 participants completed the study. Out of this initial sample, we excluded participants who: did not understand the instruction or did not fully complete the task; never attempted the Rebus puzzles (i.e., they always pressed ‘NO’ when they had to report if they had the solution of the problem); or did not solve any problem correctly. Further, we eliminated all those trials that had a reaction time shorter than 2s and trials for which participants gave an unspecified solution (i.e., scramble letters or typed sentences such as ‘I forgot the solution’, ‘I press the button by accident’ or gibberish words that showed lack of problem-solving attempt). After the data cleaning, a total of 343 participants (206 female, 130 male, and 7 nonbinary or genderfluid) remained (*M*_*age*_ = 33.5, *SD* = 7.9).

#### Power Analysis.

To determine the required sample size for our planned moderation analyses, we conducted a power analysis using G*Power 3.1 (Faul et al., 2009). Detecting interaction effects in linear regression typically requires larger sample sizes due to their relatively smaller effect sizes and increased model complexity (Aiken & West, 1991; Cohen, 1988). Assuming a small-to-medium interaction effect size (*f*^2^ = 0.025), with 80% power, and an alpha level of .05, G*Power indicated a minimum sample size of approximately 316 participants would be necessary for a model with three predictors (including the interaction term).

However, to allow for potential data exclusions (e.g., attention check failures, incomplete responses), and to ensure adequate power for detecting even slightly smaller interaction effects, we aimed for a sample size of 350 participants. This sample size also provides robustness for additional exploratory and subgroup analyses.

Our final cleaned sample included 343 participants, which exceeds the minimum required for detecting interaction effects in moderation analyses and ensures the study was well-powered to test our hypotheses involving Socio-Cognitive Polarization (SCP) as a moderator.

#### Materials.

##### Socio-Demographic Information.

A series of self-report questions assessed participant age, gender identity, marital status, education level, occupation, and country of residence.

##### Vaccine Acceptance.

Two items were used to assess vaccine acceptance: 1) the status of one’s COVID-19 vaccination (‘Are you vaccinated against COVID-19?’); 2) one’s attitude on the COVID-19 vaccine (the question, “Are you in favor of the COVID-19 vaccine?”). Participants must have demonstrated a clearly defined and coherent approach to COVID-19 vaccines to be considered for this study. To be more specific, the study only included participants whose responses to the behavioral (1) and attitudinal (2) aspects of vaccine acceptance were consistent.

##### Problem-Solving Task.

An online version of Rebus Puzzles was used to evaluate problem-solving (MacGregor & Cunningham, 2008). Participants were required to identify a common phrase using the verbal and visual clues displayed on the screen to complete the task, which was initially developed by MacGregor and Cunningham (2008). These kinds of puzzles are an established measure of insight problem solving in several online and in-person studies (e.g., Salvi, Bricolo, et al., 2016, Salvi, Costantini, et al., 2016; Salvi et al., 2020, 2021; Threadgold et al., 2018). We chose to study Rebus problem solving because the respondent must deal with multiple options and take into account multiple points of view to find a solution. To solve these puzzles, participants were instructed to identify a common phrase from the verbal and visual clues provided on screen (e.g., *‘cycle, cycle, cycle’* would be solved as *‘tricycle’*). Participants were shown 44 randomized trials, each consisting of one rebus. Each trial had a 15-second time limit.

###### Commission errors.

Two types of errors were recorded: omission errors, in which no solution was provided, and commission errors in which participants declared that they had a solution to the problem—i.e., pressed the YES button—but the solution was incorrect. The latter is used as a measure of high propositional confidence since it implies participants’ belief that they have found the correct solution to the problem when the solution is actually incorrect.

##### COVID-19 Conspiracy Theories Questionnaire.

We created an ad hoc questionnaire of 13 items based on the most common conspiracy theories percolating in the Spring of 2021 patterned after (Bertin et al., 2020). People were asked to report their agreement on the statements on a 5-point Likert scale. In accordance with former research that has emphasized the theoretical and empirical relevance of distinguishing between national ingroup and outgroup conspiracy beliefs (e.g., Cichocka et al., 2016) and because of the wide variety of COVID-19 conspiracy theories (Van Bavel et al., 2022), we designed items tapping into three group-based categories: conspiracy theories involving a threatening foreign outgroup, namely China (three items, e.g., “The coronavirus pandemic is a strategy created by China to trigger a new economic crisis”), conspiracy theories involving unspecified outgroups (i.e., not referring to any foreign country outgroups, four items, e.g., “The coronavirus vaccine contains a secret microchip that will control us”, and conspiracy theories involving members of the national ingroup, namely, the US government (three items, e.g., “The US government uses the coronavirus pandemic to distract people from real problems in the country”). Following Bertin et al. (2020) we considered three main dimensions which were labeled “Conspiracy beliefs involving an outgroup”; “Conspiracy beliefs involving an unspecified outgroup”; “Conspiracy beliefs involving the ingroup” respectively. In addition to those dimensions, we added 3 neutral questions (i.e., that did not include conspiracy theories).

##### Socio-Cognitive Polarization.

SCP, as described by Salvi et al. (2021), was measured by averaging scores obtained in conservatism, xenophobia, and absolutism as follows:

###### Political ideology.

Two 7-point Likert scales were used to measure political ideology (Robinson et al., 1999; Salvi, Cristofori, et al., 2016). Participants were asked how much they agreed with the following statements: “I endorse many aspects of liberal political ideology” and “I endorse many aspects of conservative political ideology”. Based on the answers provided we calculated a “liberalism” value obtained by subtracting the second item score from the first one. For example, if a participant gives a 3 to “I endorse many aspects of liberal political ideology” and a 7 to “I endorse many aspects of conservative political ideology” his/her/their liberalism score would be −4. This scale provided a measure of participants’ polarization on a continuum. For example, people who would score between −4 and +4 would be considered moderates, while those who score between −6/−5 and +5/+6 would be considered polarized.

###### Xenophobia.

We measured hostility and fear toward immigrants using the 14-item Xenophobia Scale created by van der Veer et al. (2013). Subjects indicated their level of agreement with statements such as “Interacting with immigrants makes me uneasy” on a 7-point Likert scale (1 = Strongly disagree; 7 = Strongly agree).

###### Absolutism.

To measure absolutism, the 30-item version of the Multidimensional Attitude Toward Ambiguity Scale (MAAS; Lauriola et al., 2016) was used. Participants were asked to rate their tolerance vs. intolerance of ambiguous stimuli (e.g., ‘There’s a right way and a wrong way to do almost everything’) on a 7-point Likert scale.

#### Procedure.

**Figure 3.**
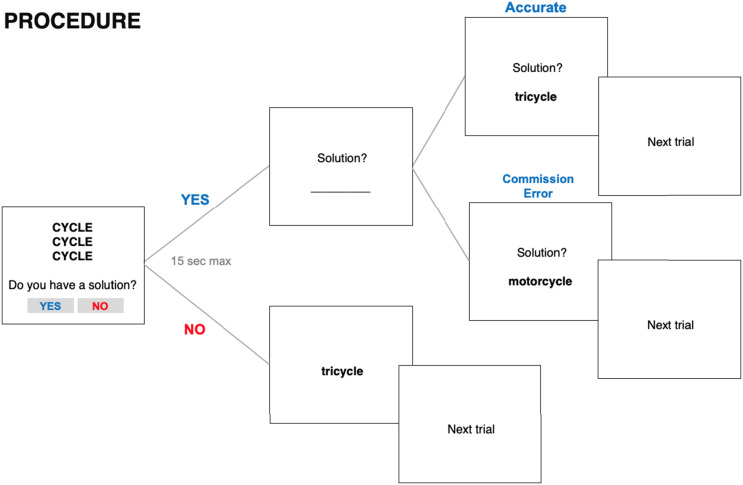
Procedure for the problem-solving section of Study 2. *Note*. Participants were given 15 seconds to solve each rebus puzzle. If they found a solution, participants had to press the YES button. Afterward, they were asked to type the solution phrase manually. If the solution was correct, it was then calculated as accurate. If it was not correct it was calculated as a “commission error”. If participants claimed that they did not have the solution, they were asked to press the NO button. If participants ran out of time, the solution would appear on the screen.

Participants first completed the demographic questionnaire, the SCP, and vaccine acceptance questions. Then, following a short practice of one trial, participants were asked to attempt solving 44 Rebus puzzles (MacGregor & Cunningham, 2008). Participants had 15 seconds to solve each puzzle within which they had to indicate if they had found the solution of the Rebus. If they thought they had a solution, they were asked to press the YES button. No feedback was provided on the accuracy of their solution. If the solution was correct, we referred to it as “problem-solving accuracy”; if the solution was incorrect, we referred to it as “problem-solving commission errors”. If participants did not have a solution to the Rebus they pressed the NO button (or the trial would time out after 15 s) after which the problem solution would immediately appear on-screen for 1 s (see Figure 3 for details).

### Results

Data analysis was performed using JASP version 19.3 (JASP Team, 2025) and the significance level was set to *p* < 0.05. Participants declared they had a solution to problems (i.e., pressed the YES button regardless of its accuracy) for an average of *M*_nop_^4^ = 21.5 (*SD*_nop_ = 5.9) problems, which corresponds to an average (i.e., *M*_nopsolved/tot problem given_^5^) of 49.1%, (*SD* = 13.6%) of problems.

Participants solved correctly (i.e., pressed the YES button and the solution was correct) for an average of *M*_nop_ = 13.9 (*SD*_nop_ = 6.1) problems, which correspond to an average (i.e., *M*_nopsolved/tot problem given_) of 30.5% (*SD* = 14.9%) of problems. Further, they assumed the solution was correct while incorrect (i.e., *Commission errors*—pressed the YES button and the solution was incorrect) for an average of *M*_nop_ = 7.6 (*SD*_nop_ = 5.2) problems, which corresponds to an average (i.e., *M*_nop/tot problem given_) of 17.3%, (*SD* = 12%) of problems. The average of participants’ omission errors (i.e., pressed the NO button) corresponds to *M*_nop_ = 22.34 (*SD*_nop_ = 5.9) of problems, which corresponds to an average (i.e., *M*_nop/tot problem given_) of 50% (*SD* = 13.6%) of problems.

#### Correlations.

Correlations are displayed in Table 2. Results show significant positive correlations among conspiratorial beliefs (both ingroup and outgroup) and SCP (xenophobia, absolutism, and conservatism) and problem-solving accuracy and problem-solving commission errors. As the table shows, conspiracy beliefs (both ingroup and outgroup) and SCP are negatively correlated with problem-solving accuracy and commission errors. Further, years of education were mildly negatively correlated only with SCP (*r* = −.14, *p* < .001).

**Table 2.** Correlation Matrix of Measures Used in Study 2. See the Supplementary Materials for a description of variables 1, 2, and 3.

**Variable**	**1**	**2**	**3**	**4**	**5**	**6**	**7**
**1. Conspiracy**	–						
**2. Conspiracy Ingroup**	0.95***	–					
**3. Conspiracy Outgroup**	.96***	.82***	–				
**4. SCP**	.66***	.62***	.65***	–			
**5. Problem Solving Accuracy**	−.36***	−.27***	−.40***	−.36***	–		
**6. Problem-Solving Commission Errors**	.24***	.16***	.29***	.22***	−.53***	–	
**7. Problem Solving Omission Errors**	.095	.076	.10	.12*	−.50***	−.36***	–

* *p* < .05.

** *p* < .01.

*** *p* < .001.

#### Moderation Analysis.

Linear regression analyses showed that problem-solving accuracy significantly predicted conspiracy beliefs, *b* = −0.36, *SE* = 0.32, *t*(341) = −7.05, *p* < .001. When Socio-Cognitive Polarization (SCP) was included as a moderator, the interaction term was significant, *b* = 0.61, *SE* = 0.011, *t*(340) = 14.35, *p* < .001, and the model explained 45% of the variance in conspiracy beliefs, *R*^2^ = .45.

Problem-solving commission errors also significantly predicted conspiracy beliefs, *b* = 0.24, *SE* = 0.010, *t*(341) = 4.65, *p* < .001. The interaction with SCP was again significant, *b* = 0.64, *SE* = 0.010, *t*(340) = 15.53, *p* < .001, with the model accounting for *R*^2^ = .45 of the variance.

In contrast, omission errors did not significantly predict conspiracy beliefs, *b* = 0.095, *SE* = 0.009, *t*(341) = 1.75, *p* = .080. However, SCP significantly moderated this relationship as well, *b* = 0.66, *SE* = 0.010, *t*(340) = 16.19, *p* < .001, with the model explaining *R*^2^ = .44 of the variance.

#### Exploratory Analyses.

**Figure 4.**
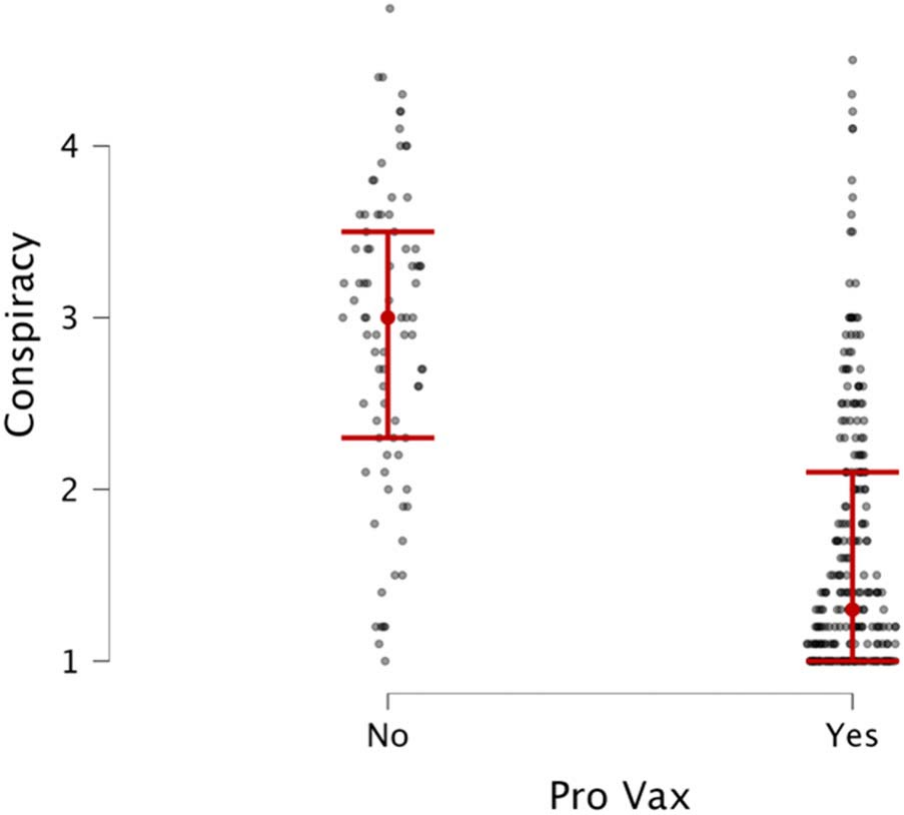
Distribution of conspiratorial beliefs between people in favor of vs. against COVID-19 vaccinations. The *x* axis maps the response to the question: “Are you in favor of the COVID-19 vaccine?”.

Further analysis revealed that people in favor of vaccines (*n* = 259) were less likely to believe in conspiracy theories, *t* = 12.6, *p* < .001, *d* = 1.57, than those not in favor of vaccines (*n* = 84) (Figure 4).

**Figure 5.**
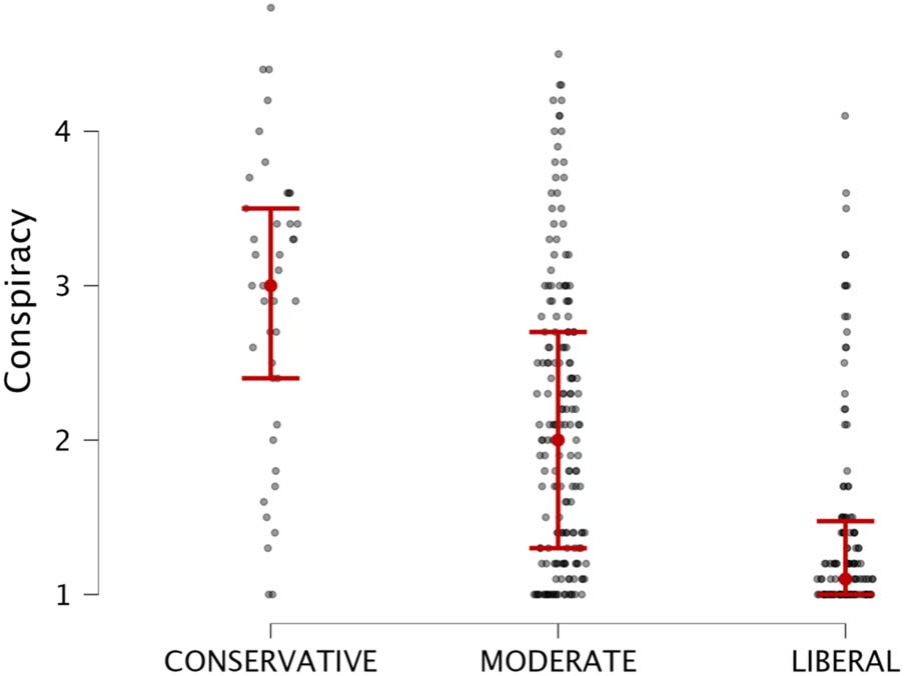
Distribution of conspiratorial beliefs across the conservative-moderate-liberal continuum.

We also explored if political polarization is associated with greater beliefs in conspiracy theories, thus we subtracted the score given on conservatism from the score given on liberalism (−6/+6 range) and we divided our sample into 3 groups: extreme conservatives (*n* = 41; score < −4); extreme liberals (*n* = 122; score > 4) moderates (*n* = 180; score between −4 and +4). As shown in Figure 5, ANOVA analysis of variance revealed a significant difference between the 3 groups and beliefs in conspiracy theories, *F*(2, 340) = 53.2, *p* < .001, *η*^2^ = .23. Post-hoc tests (using Bonferroni correction for multiple comparisons) showed a significant difference between conservatives and liberals (*t* = 9.72, *p* < .001; mean diff = 1.47; CI_95%_ = [1.11, 1.8]), conservatives and moderates (*t* = 5.42, *p* < .001, mean diff = .78; CI_95%_ = [.44, 1.13]) and liberals and moderates (*t* = −6.96, *p* < .001; mean diff = −.68; CI_95%_ = [−.91, −.45]).

### Discussion

Our findings reveal a complex interplay between problem-solving abilities, socio-cognitive polarization (SCP), and conspiracy beliefs. Correlation results showed significant positive relationships between conspiratorial beliefs and SCP dimensions (xenophobia, absolutism, and conservatism), suggesting that cognitive rigidity and polarized thinking are closely linked to conspiracy belief systems.

Based on our hypotheses, the results demonstrate several key insights. First, problem-solving accuracy was significantly negatively correlated with conspiracy beliefs, suggesting that individuals with lower problem-solving skills are more susceptible to conspiratorial thinking (see H1). This is particularly evident in the strong negative correlations between problem-solving accuracy and both ingroup and outgroup conspiracy belief measures.

Second, the moderation analyses provided crucial insights into the mechanisms underlying conspiracy belief formation. We found that problem-solving accuracy had a significant direct negative effect on conspiracy belief endorsement, with this relationship becoming even stronger when moderated by Socio-Cognitive Polarization (SCP) (see H3). Similarly, commission errors in problem solving showed a significant positive direct effect on conspiracy beliefs, which was further amplified by SCP (see H2 and H3). By contrast, omission errors did not show a significant direct relationship with conspiracy beliefs; however, SCP still significantly moderated this association, revealing that individuals higher in SCP were more susceptible to the influence of these errors.

Our exploratory analyses provided deeper insights into factors contributing to conspiracy beliefs. Notably, individuals in favor of COVID-19 vaccines were significantly less likely to endorse conspiracy theories, highlighting the potential role of scientific literacy and trust in institutional sources of knowledge as protective factors.

The political ideology analysis revealed a striking pattern: extreme conservatives demonstrated the highest levels of conspiracy beliefs, with significant differences observed between conservative, liberal, and moderate groups. This finding underscores the intricate relationship between political polarization and susceptibility to conspiratorial thinking. These results extend our understanding of conspiracy beliefs by demonstrating that they arise from a complex interplay of problem-solving skills, cognitive flexibility, and socio-political orientation. This pattern suggests that a metacognitive bias, characterized by misplaced certainty in one’s reasoning, serves as a significant cognitive underpinning of conspiratorial ideation.

Our findings reveal a complex interplay between problem-solving abilities, SCP, and conspiracy beliefs. Correlational analyses showed significant positive relationships between conspiratorial beliefs and SCP dimensions (xenophobia, absolutism, and conservatism), suggesting that cognitive rigidity and polarized thinking are closely linked to endorsement of conspiracy belief systems.

## GENERAL DISCUSSION

Conspiracy theories have persisted throughout history, influenced by political, social, and psychological factors (Lewandowsky et al., 2012; van Prooijen & Douglas, 2017). Their significant impact on public opinion and behavior, as observed during the COVID-19 pandemic, underscores the urgent need for a comprehensive investigation. This research sought to explore the cognitive and socio-cognitive factors underlying conspiracy beliefs by examining propositional confidence in metacognitive reasoning and its interaction with socio-political orientations. To this end, we created two problem-solving scenarios in which we measured participants’ predictions about their likelihood of solving unsolvable problems (initial judgment of solvability—Study 1), problem-solving accuracy, and commission errors (Study 2).

While Studies 1 and 2 utilized distinct paradigms and investigated different facets of propositional confidence, their findings collectively underscore the multifaceted nature of conspiratorial thinking evidencing how these biases play a crucial role in conspiracy beliefs.

The integration of findings from Studies 1 and 2 advances our understanding of conspiratorial thinking in several ways. First, it emphasizes that conspiracy beliefs are not solely the product of isolated cognitive fallacies but emerge from a dynamic interplay between metacognition, propositional confidence, and socio-political polarization. Second, the use of diverse problem-solving tasks—CRA problems and Rebus puzzles—highlights the generalizability of these cognitive phenomena across different domains. Finally, the incorporation of SCP in Study 2 provides a broader socio-political framework that contextualizes the findings within real-world ideological divides.

Conspiracy theorists are distinguished by a pseudo-understanding which is rooted in apophenic reasoning, whereby they construct meaning from random connections and display unwarranted certainty about intricate explanations (Vitriol & Marsh, 2018). This phenomenon suggests that in metacognitive reasoning, participants who endorse conspiracy beliefs may experience a discrepancy between their metacognitive confidence during initial impressions of problems and the actual accuracy of these impressions. In our first study, we explored this possibility by having participants make an initial judgment of solvability. Participants were shown both solvable and unsolvable CRA problems and asked to predict their likelihood of solving each problem before attempting to solve them. We observed that people who were more confident in their ability to solve the CRA problems (as indicated by their predictive judgments) tended to have higher COVID conspiracy beliefs—and this relationship was significant only for unsolvable problems. This suggests that high propositional confidence, characterized by misplaced certainty in one’s reasoning, is a significant underpinning of conspiratorial ideation. Importantly, this study laid the foundation for investigating how during metacognitive control misjudgments can manifest in belief systems that resist empirical correction.

Building on these insights, Study 2 extended the exploration to a different propositional confidence aspect—commission errors—and introduced the concept of SCP as a moderating factor. Using Rebus puzzles, Study 2 revealed that first there is a negative relationship between problem-solving accuracy and beliefs in conspiracy theories, and further and foremost that individuals prone to presuming correctness in their problem-solving were more likely to endorse conspiracy beliefs. Crucially, SCP amplified this relationship, suggesting that socio-political rigidity intertwines with cognitive biases to reinforce conspiratorial thinking. The results of Study 2 were conceptually consistent with those of Study 1. People who showed a higher percentage of commission errors were more likely to endorse conspiracy beliefs. These findings align with previous research demonstrating a negative relationship between belief in conspiracy theories, deductive reasoning, and problem-solving abilities (e.g., Sebalo et al., 2023; Swami & Furnham, 2014; van Prooijen & van Vugt, 2018).

In both studies, we found that higher problem-solving accuracy was associated with lower conspiracy beliefs. This finding is consistent with previous research showing an inverse relationship between problem-solving abilities and various measures of reasoning involving social content, such as believing in fake news and vaccine hesitancy (Cancer et al., 2023; Čavojová et al., 2020; George & Mielicki, 2023; Pennycook et al., 2015, 2020; Pennycook & Rand, 2019; Salvi, Barr, et al., 2023; Salvi, Iannello, et al., 2023; Salvi et al., 2021). Together, the studies reveal a continuum of cognitive processes that influence conspiracy beliefs. Study 1 highlights the initial cognitive miscalibrations that foster unwarranted certainty, while Study 2 demonstrates how these biases interact with socio-political dispositions to entrench belief systems. In our second study, indeed we found that the relationships between commission errors, problem-solving ability, and conspiracy beliefs were moderated by SCP. This finding highlights the complex interplay between miscalibrated meta-reasoning and socio-political factors in the formation and maintenance of conspiracy beliefs. Previous research has explored the role of metacognition in politically charged contexts (Fischer & Fleming, 2024), revealing a reduced metacognitive insight when judging politicized scientific topics, such as climate change (Fischer et al., 2019) and COVID-19 (Lisi, 2023), as compared to non-politicized domains, such as biology and physics. Moreover, although further investigation is warranted (Fischer & Fleming, 2024), the rigidity of political views appears to hinder metacognitive insight into misinformation, especially when faced with information contradicting their political views (Geers et al., 2024). Building on this work, our findings aim to advance the understanding of the meta-reasoning and socio-political underpinnings of conspiracy theories, emphasizing the importance of promoting cognitive flexibility as a means to counter misinformation and reduce susceptibility to extremist ideologies (Iannello et al., 2025).

Our findings might present an apparent contradiction: conspiracy believers demonstrate heightened pattern recognition—often seeing connections between unrelated events—yet they underperform on structured problem-solving tasks like the CRA problems. This apparent discrepancy can be explained through the lens of metacognitive monitoring and control, as outlined by Ackerman and Thompson’s (2017) dual-process meta-reasoning framework. Conspiracy believers’ enhanced pattern detection likely reflects reliance on System 1 heuristic monitoring—a fast, intuitive process that interprets familiarity or coherence as indicators of correctness. When individuals encounter partial matches or surface-level associations, System 1 generates an initial judgment of solvability, leading to an inflated sense of confidence despite lacking a genuine solution. However, tasks like CRA require System 2 analytic control, where the individual must suppress misleading associations and restructure problem elements to uncover the true solution. Conspiracy believers may struggle here because the strong subjective high propositional confidence produced by System 1 interferes with the effortful reasoning required by System 2. This leads to commission errors—mistaking partial or incorrect solutions for valid answers—perpetuating the cognitive bias that fuels conspiratorial beliefs.

Importantly, this heuristic-driven pattern detection may offer an adaptive advantage in uncertain or ambiguous environments, where rapid detection of connections could enhance perceived understanding. However, in structured tasks requiring flexible, analytic restructuring (e.g., CRA problems), this same cognitive shortcut becomes a liability, fostering errors rather than insights.

By integrating these insights with Ackerman and Thompson’s (2017) meta-reasoning framework, we propose that conspiracy believers’ pattern-seeking behavior stems from an imbalance between heuristic monitoring and analytic control. Their tendency to over-rely on System 1 not only increases false confidence but also inhibits the corrective processes that might otherwise help them recognize errors—contributing to both poor problem-solving performance and persistent belief in conspiratorial explanations.

Prior studies have consistently found that individuals with higher beliefs in conspiracy theories have lower metacognitive control and poorer problem-solving performance (Douglas et al., 2017). For example, Swami and Furnham’s (2014) seminal work identified analytical thinking as a measurable cognitive approach that can reduce conspiracist ideation. Promoting analytical cognitive styles has been proposed as a method to counter conspiracy theories (Orosz et al., 2016; van Prooijen & Douglas, 2017). Studies have also found inverse relationships between problem-solving ability and various measures of social reasoning, including belief in fake news and vaccine hesitancy (Cancer et al., 2023; Salvi, Barr, et al., 2023; Salvi, Iannello, et al., 2023; Salvi et al., 2021; Zmigrod, 2020; Zmigrod et al., 2018, 2019). Some researchers suggest that the “crippled epistemology” characteristic of political polarization is also present in conspiracy beliefs (Sunstein & Vermeule, 2009; Swami & Furnham, 2014), potentially due to a highly structured thinking style aimed at making sense of societal events (Fernbach et al., 2013; Greenberg & Jonas, 2003; Kruglanski et al., 2006).

Our exploratory analyses revealed that individuals in favor of COVID-19 vaccines were significantly less likely to endorse conspiracy theories, highlighting the role of scientific literacy and trust in institutional knowledge. These findings suggest that fostering scientific understanding and promoting institutional trust could serve as protective factors against conspiratorial thinking. Furthermore, the political ideology analysis revealed a striking pattern: extreme conservatives demonstrated the highest levels of conspiracy beliefs, with significant differences observed between conservative, liberal, and moderate groups. This finding underscores the intricate relationship between political polarization and susceptibility to conspiratorial thinking. By illustrating how political ideology intersects with cognitive and metacognitive processes, these results highlight the need for strategies that address ideological polarization as a means of reducing conspiracy beliefs. Such interventions could include fostering open-mindedness and critical thinking across the ideological spectrum to mitigate the effects of extreme political ideologies on belief in conspiracy theories.

## CONCLUSION

Across two studies, we demonstrated that propositional confidence—specifically initial judgment of solvability and commission errors—are key predictors of conspiracy beliefs. Individuals who overestimate their problem-solving abilities, particularly in the face of unsolvable tasks, are more likely to endorse conspiratorial thinking.

Moreover, we addressed the paradox of conspiracy believers’ heightened pattern recognition. While they excel at detecting superficial connections between unrelated events, this heuristic-driven pattern detection fuels false certainty. This, in turn, reduces problem-solving accuracy on structured tasks that require analytical flexibility—highlighting how surface-level associations can masquerade as genuine insights.

Crucially, we found that SCP moderates these biases. SCP reinforces reliance on heuristic reasoning while weakening analytic control, creating an environment where propositional confidence and pattern misinterpretations thrive. This interplay helps explain why conspiracy beliefs remain resilient to correction, even when individuals consistently generate incorrect solutions.

Together, these findings underscore the complex interaction between metacognitive processes and socio-political attitudes. Future research and interventions may benefit from targeting both cognitive biases and the ideological frameworks that sustain them, fostering more reflective, flexible thinking to combat conspiratorial belief systems.

## LIMITATIONS AND FUTURE DIRECTIONS

While this research provides valuable insights into the relationship between metacognitive inaccuracies, problem-solving abilities, and conspiracy beliefs, several limitations warrant consideration.

Our reliance on self-reported measures for conspiracy beliefs may have introduced biases, particularly social desirability bias. Participants might have underreported their beliefs in socially sensitive conspiracy theories to align with perceived social norms. Future studies could address this limitation by incorporating implicit measures or behavioral indicators of conspiracy belief, which may provide a more nuanced understanding of these attitudes.

Both CRA problems and Rebus puzzles served to examine meta-reasoning propositional confidence across diverse domains—purely verbal in the case of CRA and a mix of pictorial and verbal elements in Rebus puzzles. This methodological diversity helps rule out task-specific effects and enhances the generalizability of our findings. However, the differing content and cognitive demands of these tasks may introduce task-specific effects that could influence results. Future research should strive to further validate these findings using additional problem types to ensure robustness across contexts.

We acknowledge that while our Study 2 design captured whether participants made commission errors, it did not include an explicit confidence rating for each response. This is a limitation, as prior research (e.g., Stuyck et al., 2021) shows that incorrect responses are often associated with lower confidence, and participants may knowingly submit false answers which complicates the interpretation of commission errors as purely metacognitive failures (Salvi, Bricolo, et al., 2016). Further, we acknowledge that commission errors may partly reflect task difficulty rather than certainty *per se*. While previous work (e.g., Kiani & Shadlen, 2009) has interpreted opt-out choices as indicators of confidence—showing improved accuracy when agents can opt-out—the analysis of omission errors did not show a correlation with conspiracy theories (only with SCP). We therefore interpreted only commission errors as suggestive of misplaced certainty but note that this is an indirect measure. To more accurately assess metacognitive monitoring, future studies should incorporate direct confidence judgments alongside solution submissions, allowing for clearer differentiation between errors driven by overconfidence and those made with uncertainty or strategic guessing.

This research focused on COVID-19 conspiracy theories during the pandemic, a time of unprecedented global uncertainty and rapid social change. The dynamic nature of the pandemic—including political shifts and vaccine availability—necessitated the use of scales tailored to the most prevalent conspiracy theories at two distinct time points. While this approach ensured relevance, it may limit the generalizability of our findings to other types of conspiracy beliefs. Future studies should aim to examine more stable, cross-contextual conspiracy beliefs to enhance the applicability of findings.

Both studies were conducted with U.S. participants, potentially restricting the cross-cultural applicability of our results. Cultural factors, such as political systems, societal norms, and historical contexts, may influence conspiracy beliefs and their cognitive correlates. Further research is needed to explore these relationships in diverse cultural settings to determine their universality.

Further, we acknowledge that the sample size in Study 1, while sufficient for detecting medium effect sizes, may not have been optimal for identifying smaller effects. A power analysis using G*Power indicated that detecting a medium correlation (*r* = .30) with 80% power at *α* = .05 required at least 84 participants and detecting a medium effect size in multiple regression (*f*^2^ = 0.15) with two predictors required 68 participants. Our final sample of 100 exceeded both thresholds, allowing for reliable detection of medium to small effects. However, this size still limits our ability to detect small correlations (e.g., *r* = .10–.20) with adequate power, which could be meaningful in the context of complex psychological phenomena such as metacognition and conspiracy beliefs. Thus, while our sample meets accepted standards and aligns with recent recommendations in individual differences research (Gignac & Szodorai, 2016), future studies with larger samples may be necessary to uncover more subtle relationships.

Further, given the evolving nature of conspiracy beliefs, especially during crises like the pandemic, a longitudinal design could have offered valuable insights into how these beliefs develop and change over time. Future research should consider employing longitudinal methodologies to capture the dynamic interplay between cognitive biases and socio-political factors.

Despite these limitations, this study makes significant contributions to understanding the cognitive and socio-political underpinnings of conspiracy beliefs. By highlighting the role of meta-reasoning and problem-solving abilities, it lays a robust foundation for future investigations and interventions aimed at mitigating conspiracy thinking.

## ACKNOWLEDGMENTS

We thank Simona Sacchi for her valuable comments on the analysis of Experiment 2. We are also grateful to Fabrizio Conti for providing an insightful quote on witches. Finally, we thank the reviewers for their supportive and constructive feedback, which greatly helped improve the manuscript.

## FUNDING INFORMATION

This research was supported by institutional resources; no external funding was received.

## AUTHOR CONTRIBUTIONS

C.S.: Conceptualization; Formal analysis; Methodology; Supervision; Writing – original draft; Writing – review & editing. T.G.: Conceptualization; Formal analysis; Methodology; Supervision; Writing – original draft; Writing – review & editing.

## DATA AVAILABILITY STATEMENT

All data supporting the findings of this study are available from the corresponding author upon reasonable request.

## Notes

^1^ That is, claiming of having the solution of a problem when it is incorrect. Commission errors reflect overconfident or misguided attempts to produce solutions and can serve as a marker of how well someone monitors and controls their own thinking.^2^ That is, monitoring and control of reasoning and problem solving (Ackerman & Thompson, 2017).^3^ Due to substantial correlation between solvable and unsolvable CRAs, we note that multicollinearity diagnostics indicated acceptable levels (VIF = 2.40, Tolerance = 0.42).^4^ Number of problems (nop) per participant. Average of number of problems across participants.^5^ Percent of problems per participant, calculated on the number on the total number of given problems. For each participant, we divided the number of problems by the total problems given and averaged those.
